# *Developing inclusive youth*: children’s moral reasoning predicts inclusive orientations

**DOI:** 10.1007/s11218-025-10117-6

**Published:** 2025-08-07

**Authors:** Kathryn M. Yee, Kate Luken Raz, Riley N. Sims, Melanie Killen

**Affiliations:** Department of Human Development and Quantitative Methodology, University of Maryland, College Park, MD USA

**Keywords:** Moral reasoning, Intergroup attitudes, Social exclusion, Empathy, Inclusion

## Abstract

This study examined how children’s moral reasoning in response to intergroup exclusion scenarios relates to inclusive attitudes and behaviors. A sample of 528 students (*M*_*age*_ = 9.19, *SD* = 0.90; 264 girls) in third through fifth grade participated in the *Developing Inclusive Youth* (DIY) program, which provided structured opportunities for moral reasoning through varied intergroup scenarios and peer discussions. Results showed that more frequent use of moral reasoning predicted greater inclusivity across multiple measures. Children who engaged in higher levels of moral reasoning demonstrated more negative evaluations of exclusion, greater empathy toward peers from multiple racial groups, and a stronger desire to play with those peers. However, moral reasoning was not significantly associated with expectations for inclusion or with attitudes toward boys or girls. No significant interactions emerged between moral reasoning and participant demographics (race, gender, grade), suggesting broadly applicable effects. These findings highlight moral reasoning as a key mechanism for promoting inclusive orientations in childhood, particularly in contexts involving racial diversity. Future research should explore how moral reasoning interacts with other factors, such as empathy, perspective-taking, and group norms, to support inclusivity across social contexts.

## Introduction

Childhood is a critical period for the development of social attitudes, when tendencies related to group membership, such as preferences, stereotypes, and early forms of exclusion begin to emerge yet remain malleable (Rutland et al., 2024). As such, promoting inclusivity during this period is both timely and foundational for long-term social development. Recent societal shifts have underscored the importance of addressing exclusionary behaviors and attitudes early in life.

Children today are growing up in social environments where they are routinely exposed to group-based differences in treatment, including those related to race and ethnicity, gender, religion, nationality, and socioeconomic status. These dynamics can shape children’s peer relationships, perceptions of fairness, and sense of belonging from an early age. At the same time, global events have intensified the salience of intergroup divisions. For instance, the COVID-19 pandemic contributed to a documented rise in anti-Asian sentiment and hate crimes (Center for the Study of Hate & Extremism, 2021), while domestic and international political conflicts have fueled polarization and xenophobia (Pew Research Center, 2024). Meanwhile, global migration and demographic shifts have led to increasing racial, ethnic, and cultural diversity in schools (National Center for Education Statistics, 2019), presenting both challenges and opportunities for fostering inclusive peer environments. Wealth inequality continues to disproportionately affect marginalized racial and ethnic groups, immigrants, and low-income students, reinforcing disparities in educational and social outcomes (Bécares & Priest, 2015).

These societal tensions do not exist in a vacuum for children. Rather, children actively interpret exclusion, inequality, and group-based differences through their developing cognitive, social, and moral reasoning skills (Killen & Dahl, 2021). Early social preferences, such as favoring familiar or ingroup members, are normative in childhood and support trust, affiliation, and cooperation (Fawcett & Markson, 2010; Richter et al., 2016; Sparks et al., 2017). However, when unexamined, these preferences can also reinforce social divisions by contributing to stereotyping, prejudice, and exclusion (Rivas-Drake et al., 2014; Rutland et al., 2024). These consequences are not merely interpersonal. Exclusion and discrimination during childhood are associated with adverse outcomes, including lower self-esteem, heightened depressive symptoms, and disengagement from academic contexts (Benner et al., 2018; Mendes et al., 2007). Conversely, meaningful cross-group contact can enhance empathy, reduce intergroup anxiety, and promote more inclusive peer dynamics (Brown & Paterson, 2016; Tropp & Barlow, 2018).

Thus, developmental science has increasingly focused on understanding the mechanisms that promote inclusive behavior and reduce bias during childhood. One such mechanism is moral reasoning, or the capacity to evaluate social situations in terms of fairness, rights, and concern for others’ welfare. As children encounter exclusion and group-based bias in their daily lives, the extent to which they rely on moral considerations, versus group-based or pragmatic concerns, may shape their inclusive orientations and behaviors. Examining how frequently and in what contexts children prioritize moral reasoning provides key insight into how they make sense of intergroup dynamics and informs intervention approaches designed to foster inclusivity.

### Moral reasoning and prejudice reduction

The Social Reasoning Developmental (SRD) model posits that in intergroup situations, children weigh moral considerations, such as fairness, others’ welfare, and rights, against group-based concerns, including stereotypes, group functioning, and dynamics like ingroup preference and norms (Killen & Rutland, 2011). As children develop a more complex understanding of group dynamics, their evaluations of social situations become increasingly nuanced (Elenbaas et al., 2020). For instance, when deciding whether to include or exclude a peer, children may simultaneously consider what is fair, how the peer would feel if included or excluded, how the decision might affect other group harmony, and how it might impact their own social standing. These concerns are further shaped by contextual factors, such as age and environment, which influence how children prioritize different concerns in a given situation. With age, children also develop more sophisticated awareness of societal structures and power dynamics, which may lead them to rationalize exclusion through non-moral lenses, such as perceived ingroup cohesion or practical group functioning (Rutland et al., 2024).

Moral reasoning—the process by which individuals adhere to prescriptive principles related to others’ welfare, rights, equality, fairness, and justice—has been associated with a reduction in prejudicial attitudes (Killen & Dahl, 2021; Turiel, 2015). Engagement in such reasoning fosters a sense of moral responsibility, prompting children to recognize and address disparities and injustices in their social environments (Wainryb & Recchia, 2014). Children who consistently prioritize moral principles are more inclined to engage in actions that promote fairness and equality, such as intervening in bullying, supporting marginalized peers, or advocating for inclusive practices in their schools (Killen & Dahl, 2021). These behaviors not only reflect a commitment to justice but also contribute to the creation of a more inclusive and supportive social climate.

However, adhering to moral principles is not always automatic. Children often experience tension between moral principles and other concerns, such as conformity to group norms or personal interests (Rutland & Killen, 2015). For instance, a child may recognize that excluding a peer based on their race or gender is wrong but hesitate to act due to fear of social repercussions or a desire to fit in with their peer group. This tension underscores the complexity of children’s everyday social interactions and the need to understand how they navigate these competing concerns. Children vary considerably in how they prioritize different types of reasoning in real-world situations. Understanding how programs can support the application of moral reasoning in intergroup contexts remains critical.

### Intergroup contact and inclusion

Intergroup contact theory offers one promising approach for reducing prejudice by facilitating positive interactions between members of different social groups, particularly when supported by authority figures (Feddes et al., 2009; Tropp & Prenovost, 2008). Intergroup contact reduces prejudice through multiple mechanisms. First, it increases knowledge about the outgroup, helping children learn about different cultural norms, values, and perspectives, which can dispel stereotypes. Second, it reduces the anxiety that children often feel during intergroup encounters by normalizing interactions with diverse peers. This reduction in anxiety fosters a sense of comfort and ease, making future interactions more positive. Third, intergroup contact enhances empathy and perspective-taking by encouraging children to see the world through the eyes of others, promoting emotional connections and understanding.

When direct contact is not feasible, indirect forms of contact, such stories, videos, or imagined interactions, can also significantly shape children’s perceptions of social norms (Brown & Paterson, 2016). For instance, hearing a fictional story about a peer who shares their identity being friends with someone of a different identity can increase perceptions that positive intergroup contact is normative. Simply perceiving that positive contact with outgroups is commonplace can reduce negative outgroup attitudes (Christ et al., 2014). In school settings, where diverse groups of children interact under the guidance of teachers, both direct and indirect intergroup contact present powerful opportunities to promote inclusion.

Moreover, children’s interpretations of intergroup contact and exclusion often vary by their own group memberships. For example, White children tend to view same-race inclusion as more normative than interracial inclusion, whereas Black children perceive both same-race and interracial inclusion as equally likely (Shutts et al., 2013). Additionally, children from disadvantaged groups (e.g., girls, racial minorities) are more likely to evaluate exclusion negatively and demonstrate stronger perspective-taking abilities than their advantaged counterparts (Rizzo & Killen, 2018). These patterns suggest that children’s reasoning about inclusion and exclusion is shaped not only by developmental stage but also by their own social identities and experiences.

Despite this growing knowledge, few studies have examined how children’s moral reasoning about intergroup exclusion scenarios involving diverse identities translates to broader inclusive behaviors and attitudes. Even fewer have explored whether these associations vary by children’s demographic characteristics across contexts where direct and indirect forms of intergroup contact are facilitated. The current study addresses these gaps by examining children’s use of moral reasoning during a structured program and its relations to their inclusive orientations toward diverse peers, including empathy, desire for contact, expectations for inclusion, and evaluations of exclusion. By integrating insights from developmental science and intergroup contact theory, this study seeks to explore how moral reasoning operates as a mechanism for promoting inclusion in childhood.

## The current study

The current study aimed to extend prior research by examining how children’s use of moral reasoning when responding to intergroup exclusion scenarios involving diverse social identities (e.g., race, ethnicity, gender, socioeconomic status) was associated with their broader inclusive attitudes and behaviors. Specifically, we investigated whether the frequency of moral reasoning expressed while participating in the Developing Inclusive Youth (DIY) program (Killen et al., 2022) predicted children’s evaluations of exclusion, expectations for inclusion, desire to play with diverse peers, and empathy toward diverse peers. In this study, we use the term “racially-diverse peers” to refer specifically to White, Black, and Asian children depicted in the illustrated stimuli included in the outcome measures, and “gender-diverse peers” to refer to boys and girls shown in those same measures.

The DIY program was designed to provide structured opportunities for both indirect and direct intergroup contact, drawing on prior research indicating the efficacy of these forms of contact in reducing bias and fostering inclusivity (Brown & Paterson, 2016; Tropp et al., 2022). Through a series of structured scenarios and guided peer discussions, the program aimed to promote perspective-taking, empathy, and reflection on fairness and inclusion. These sessions offered children exposure to diverse characters and opportunities to engage in dialogue with peers about intergroup exclusion, providing both vicarious and real-world contexts to support the development of inclusive attitudes and behaviors.

Our primary hypothesis was that children who more frequently applied moral reasoning during the eight-week program would report more negative evaluations of exclusion, higher expectations for inclusion, greater desire to play with diverse peers, and greater empathy toward diverse peers. Building on prior research showing that moral reasoning supports prosocial behaviors and reduces intergroup biases (Killen & Dahl, 2021), we further anticipated that these associations would be particularly pronounced in interracial contexts, where concerns about fairness and justice are often heightened (Rutland & Killen, 2015).

In addition, we conducted exploratory analyses to examine potential interactions between children’s moral reasoning and participant demographics (race, gender, and grade). Although we did not have specific hypotheses for these interactions, prior research suggests they are plausible. For instance, younger children may be more likely to view exclusion as unjust, relying primarily on moral reasoning, whereas older children may increasingly consider group functioning concerns, resulting in more nuanced or mixed evaluations (Mulvey, 2016). Moreover, program effects may vary by participant race, with some studies suggesting that programs are often more effective for majority-group children (Beelmann & Heinemann, 2014). Children who are disadvantaged by race, gender, or socioeconomic status may also be more likely to reject exclusion and exhibit greater use of moral reasoning (Burkholder et al., 2020; Rizzo & Killen, 2020). Exploring these interactions allowed us to examine whether the associations between moral reasoning and inclusivity generalize across diverse groups or vary based on children’s social identities and developmental stage.

## Method

### Participants

The data reported here are a subset from a randomized control trial evaluating the DIY intervention program among third, fourth, and fifth grades in six schools in a large Mid-Atlantic public school district (Killen et al., 2022). Within each school and grade, classrooms were randomly assigned the DIY program or a business-as-usual (BAU) control condition. The current study included students in the DIY program (*N* = 528), comprising third grade (*n* = 173, girls = 90, *M*_*age*_ = 8.63, *SD*_*age*_ = 0.34), fourth grade (*n* = 178, girls = 91, *M*_*age*_ = 9.66, *SD*_*age*_ = 0.39), and fifth grade (*n* = 177, girls = 83, *M*_*age*_ = 10.61, *SD*_*age*_ = 0.37. Participants represented both majority and minority racial and ethnic groups (see Table 1).

**Table 1:** Demographic characteristics of participants in the DIY program (*N* = 528)

Student characteristic	Percentage
Grade level	
3rd	32.77%
4th	33.90%
5th	33.33%
Gender	
Girls	50.00%
Boys	49.62%
Not reported	0.38%
Race/ethnicity	
European American	55.30%
African American	5.87%
Latinx	4.73%
Asian American	8.52%
Multiethnic	13.64%
Other	7.01%
Not reported	4.92%

*Note*. Percentages are based on parent-reported data

Following the school district and principal approvals, consent forms were distributed to all eligible classrooms. Written parental consent and verbal participant assent were obtained prior to participation. The overall parental consent rate for the full study, including both conditions, was 83.6%. Students without consent completed alternative activities during data collection and program sessions.

Participating schools had a relatively low percentage of students receiving Free and Reduced-Price Meals (*M* = 8.1%; range 5%-11.4%). Although we did not collect individual-level socioeconomic data, this suggests that the schools served predominantly economically advantaged communities. All procedures were approved by the University of Maryland Institutional Review Board [Protocol #1093717] and data collection took place during fall 2018 and fall 2019.

An a priori power analysis using G*Power (Faul et al., 2009) indicated that a sample of 316 participants would be sufficient to detect the incremental variance explained by the block of interaction terms (moral reasoning × grade, gender, and race) assuming a small-to-moderate effect size (*f*² = 0.035), *α* = 0.05, and power = 0.80. Our sample of 528 participants ensured adequate power to detect interaction effects.

### Materials and procedure

Participants completed an online questionnaire approximately one week before (pretest) and one week after (posttest) participating in the DIY program. The pretest and posttest included assessments of evaluations of exclusion, expectations for inclusion, desire to play with diverse peers, and empathy toward diverse peers. The full assessment took approximately 30 min. Between assessments, students in the DIY classrooms participated in eight weekly program sessions, each lasting approximately 45 min and consisting of two components: a web-based curriculum tool and a teacher-led classroom discussion. The present study focuses on participants’ moral reasoning during the web-based curriculum tool portion. The classroom discussions provided important context for program delivery by fostering peer dialogue about inclusion and exclusion and providing opportunities for teacher-supported direct intergroup peer contact.

#### Web-based curriculum tool

**Fig. 1:**
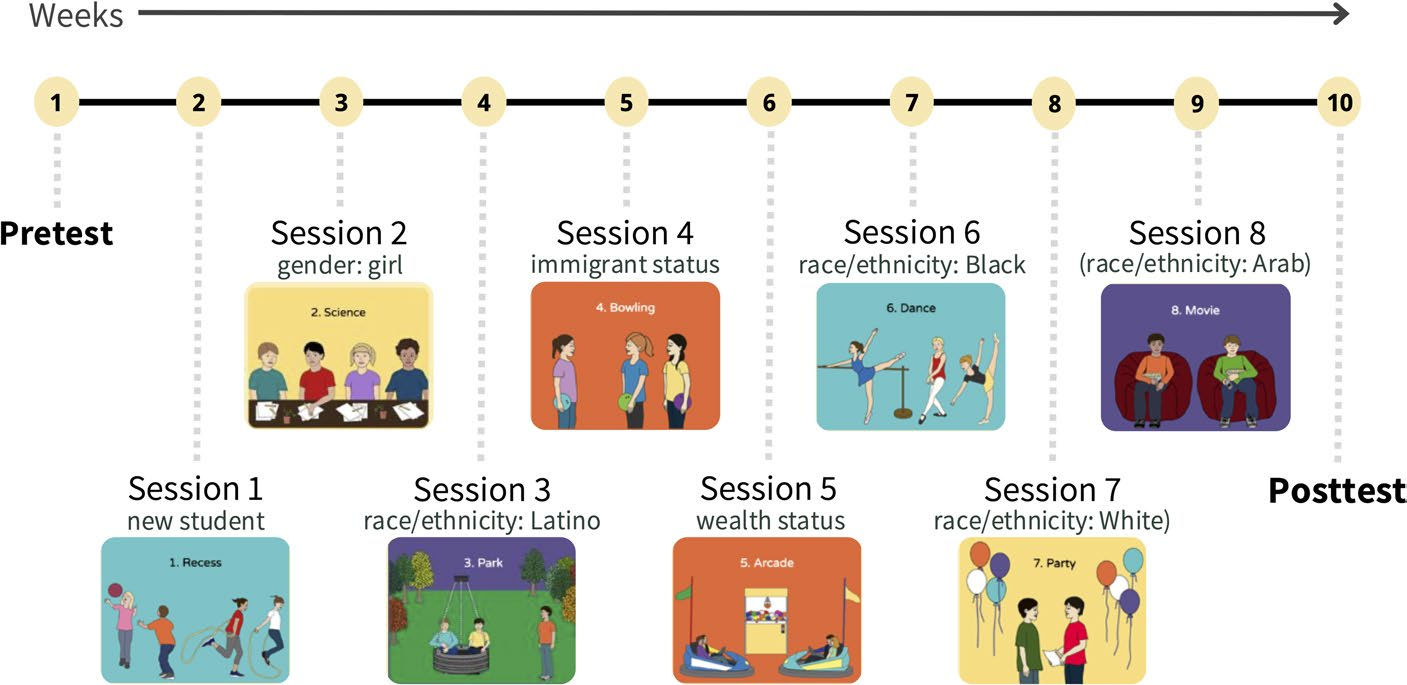
Overview of the DIY program sessions and assessments. *Note*. Illustrations copyright by Joan Tycko

During each DIY session, students individually interacted with a web-based curriculum presenting scenarios in which a peer was excluded due to their social identity. Each scenario depicted an everyday situation (e.g., school, park, home) and featured diverse targets of exclusion, including a new student, a girl, a Latino peer, an immigrant peer, a wealthy peer, a Black peer, a White peer, and an Arab American peer (see Fig. 1). Students made decisions, predicted characters’ feelings, evaluated exclusion, and provided justifications. Participants’ moral reasoning was assessed during these scenarios.

#### Teacher-led classroom discussion

Following the individual activity, students participated in a teacher-led discussion, guided by pre-training and provided materials. Teachers encouraged peer dialogue rather than direct instruction, prompting students to connect the week’s scenario to their own experiences and consider different perspectives. Although we did not systematically quantify students’ use of moral reasoning during these discussions, informal observations and selective transcripts indicated that students frequently referenced fairness, others’ welfare, and rights in reference to characters presented in the fictional scenarios as well as their personal experiences.

### Measures

Participants’ moral reasoning was assessed during the web-based curriculum tool portion of the DIY sessions over the course of the 8-week program. The four outcome measures were assessed at both pretest and posttest.

#### Moral reasoning

During each scenario presented in the DIY curriculum tool, participants evaluated the excluder’s behavior by indicating whether they thought it was “okay” or “not okay” to exclude the peer. For example, in the *Science* scenario, participants were introduced to a boy named Mark, who did not want to let a girl named Samantha join his science group, which already included two other boys. After viewing the scenario, participants were asked to evaluate whether it was “okay” or “not okay” for Mark to exclude Samantha from the group. Following their evaluation, all participants, regardless of whether they judged the exclusion as acceptable or not, were asked to select one of four reasoning options to justify their evaluation. Participants who responded that exclusion was “not okay” were presented with four reasoning choices reflecting:

- *Others’ welfare* (e.g., “She will be hurt if they don’t let her join”).
- *Fairness* (e.g., “It’s unfair to keep her out just because she’s a girl”).
- *Pragmatism* (e.g., “She might be good at science and help them get a better grade”).
- *Personal Choice* (e.g., “If Samantha wants to join, then let her do what she wants”).

Participants who judged the exclusion as “okay” were also presented with four reasoning choices reflecting *Stereotypes* (e.g., “Girls aren’t good at science”), *Ingroup Preference* (e.g., “The boys should work together, because they just like working with boys”), *Pragmatism* (e.g., “Four is too many people for a group. Three people will work better together”), and *Personal Choice* (e.g., “It’s up to them who they want to work with or not”). Thus, all participants were required to provide a justification for their evaluation regardless of whether they judged the exclusion as acceptable or unacceptable.

Moral reasoning was coded as “1” if the participant selected Other’s Welfare or Fairness and as “0” if they selected any other option. To calculate each participant’s overall use of moral reasoning, we aggregated their moral reasoning scores across the scenarios and divided this by the number of scenarios they completed (out of a possible 8). The proportion of moral reasoning scores ranged from 0 to 1 (*M* = 0.58, *SD* = 0.23), with higher values reflecting more frequent use of moral reasoning across the scenarios.

#### Evaluations of exclusion

Participants evaluated peer exclusion using four illustrated vignettes adapted from Cooley et al. (2019), in which two characters decided whether to include or exclude a peer. The vignettes varied the racial context of the encounter as follows:

1. Same-race: Two White characters consider excluding a White peer.
2. Same-race: Two Black characters consider excluding a Black peer.
3. Interracial: Two White characters consider excluding a Black peer.
4. Interracial: Two Black characters consider excluding a White peer.

Characters were gender-matched to participants. For example, in the interracial vignette shown to girls, two White characters, Jenny and Allison, considered excluding a Black peer, Rachel: *“It’s Jenny’s birthday and she’s having a party. She invited all her friends*,* including her best friend*,* Allison. She can only invite one more person and she is thinking about inviting Rachel*,* the new kid at school. Allison doesn’t think she should invite Rachel.”*

After reading each vignette, participants were asked, *“Let’s say Jenny decides not to invite Rachel because she’s worried Allison won’t like it. How okay or not okay is that?*” Responses were recorded on a 6-point Likert scale ranging from 1 (“really not okay”) to 6 (“really okay”). These scores were later reverse-coded, so that higher scores reflected more negative evaluations of exclusion.

#### Expectations for inclusion

For the same four vignettes described previously, participants also rated their expectations regarding whether the protagonist would include the peer. For each vignette, they were asked, *“How likely it is that [Protagonist] will invite the [New Kid]?”* Responses were recorded on a 6-point Likert scale ranging from 1 (“really unlikely”) to 6 (“really likely”).

#### Desire to play with diverse peers

Participants’ desire to play with gender- and racially-diverse peers was assessed using adapted measures from prior research (Aronson & Brown, 2013; Brenick & Romano, 2016). Participants were shown five illustrations depicting groups of children that varied by gender (girl, boy) or race (White, Black, Asian).

For the gender illustrations, four characters were depicted as black silhouettes to control for race. Participants were asked, *“Here are some [girls/boys]. How much would you want to play with [girls/boys]?”*

For the race illustrations, two boys and two girls representing each racial group were shown in color, with race indicated through physical features such as skin tone. Participants were asked, *“Here are some kids who look like this. How much would you want to play with kids who look like this?”*

All responses were indicated on a 6-point Likert scale ranging from 1 (“really don’t want to”) to 6 (“really want to”). Two composite scores were calculated: one for gender-related items and another for race-related items.

#### Empathy toward diverse peers

Empathy toward gender- and racially-diverse peers was assessed using the same five stimuli from the Desire to Play with Diverse Peers measure. Questions were adapted from established developmental research (Gasser et al., 2013; Grütter et al., 2018; Malti et al., 2009, p. 200).

For the gender items, participants were asked, *“Here are some [girls/boys]. How would you feel if a [girl/boy] from this group were alone most of the time in school?”* For the race items, they were asked, *“Here are some kids who look like this. How would you feel if a kid from this group were alone most of the time in school?”*

Responses were recorded on a 6-point Likert scale ranging from 1 (“really bad”) to 6 (“really good”). Two composite scores were calculated: one for gender-related items and another for race-related items.

## Data analytic plan

The overall attrition rate was low for our sample, with less than 4% of data missing on the outcome measures. However, providing racial demographic information was optional for parents, resulting in 10.8% missing data on participant race. Given our interest in race as a covariate and the need to minimize bias associated with listwise deletion, missing race data were handled using multiple imputation. This approach aligns with best practices for handling missing demographic data, particularly when these variables are included as covariates rather than primary outcomes (Enders, 2010; White et al., 2011).

Multiple imputations were conducted using the mice package in R, with predictive mean matching. Variables included in the imputation model were demographic characteristics (grade, classroom, gender, race, and school), along with all pretest and posttest outcome variables: evaluations of exclusion, expectations for inclusion, desire to play with diverse peers, and empathy toward diverse peers. Following Graham and Hofer’s (2000) guidelines, we created 30 imputed datasets. Analyses were performed separately on each imputed dataset, and parameter estimates and associated sampling variances were pooled to generate final model estimates.

Prior to testing our hypotheses, we conducted preliminary analyses to determine whether the classroom-level clustering should be accounted for in the models. Intraclass Correlation Coefficients (ICCs) were calculated for each outcome, and in all cases, ICCs did not exceed 0.02. Model comparisons further confirmed that including classroom as a random or a fixed effect did not improve model fit for any outcome variable. Therefore, all analyses were conducted at the individual level without adjusting for classroom clustering.

To test our primary hypothesis that children’s use of moral reasoning during the DIY program would predict more inclusive orientations toward diverse peers, we conducted multiple regression analyses for each outcome measure: evaluations of exclusion (same-race/interracial), expectations for inclusion (same-race/interracial), desire to play with diverse peers (gender/race), empathy toward diverse peers (gender/race). For each imputed dataset, posttest scores for each outcome were regressed on the respective pretest score (to control for baseline levels), moral reasoning, participant grade, gender, and race. Final estimates were derived by pooling results across the 30 imputed datasets.

## Results

To examine whether the associations between children’s use of moral reasoning and the outcome measures varied by participant demographic variables (gender, race, and grade), we conducted model comparisons across five models for each outcome. Model 1 included the main effects of the predictor variables (moral reasoning, grade, gender, and race) along with the pretest score for the respective outcome as a control. Models 2 through 4 each added one interaction term between moral reasoning and either grade, gender, or race, respectively. Model 5 included all three interaction terms simultaneously. Likelihood Ratio Tests were used to compare Model 1 with Models 2–5 for each outcome measure. Across all outcomes, model comparisons indicated that none of the interaction terms significantly improved model fit (ps > 0.05). Thus, no significant interactions between moral reasoning and demographic variables were detected, and we proceeded with Model 1 (main effects only) for all outcome analyses. A summary of the results for the primary hypotheses is presented in Table 2.

**Table 2:** Effects of moral reasoning on inclusive orientations

Outcome	$\:\beta\:$ (SE)	B	95% CI	*p*
Negative evaluation of same-race exclusion	1.07 (0.22)	0.21	[0.64, 1.49]	< 0.001*
Negative evaluation of interracial exclusion	1.40 (0.22)	0.27	[0.97, 1.83]	< 0.001*
Expected same-race inclusion	0.15 (0.22)	0.03	[-0.29, 0.58]	0.51
Expected of interracial inclusion	0.09 (0.26)	0.01	[-0.43, 0.60]	0.75
Desire to play with gender-diverse peers	0.30 (0.19)	0.08	[-0.07, 0.66]	0.11
Desire to play with racially-diverse peers	0.48 (0.21)	0.11	[0.06, 0.90]	0.03*
Empathy for gender-diverse peers	0.33 (0.22)	0.07	[-0.11, 0.76]	0.15
Empathy for racially-diverse peers	0.61 (0.23)	0.12	[0.16, 1.05]	0.008*

*Note*.* β* = unstandardized regression coefficients; *SE* = standard error; *B* = standardized regression coefficient (effect size); CI = confidence interval. Values reflect posttest outcomes controlling for pretest scores. Significant values denoted as *p <.05; ***p <.001

### Evaluations of exclusion

**Fig. 2:**
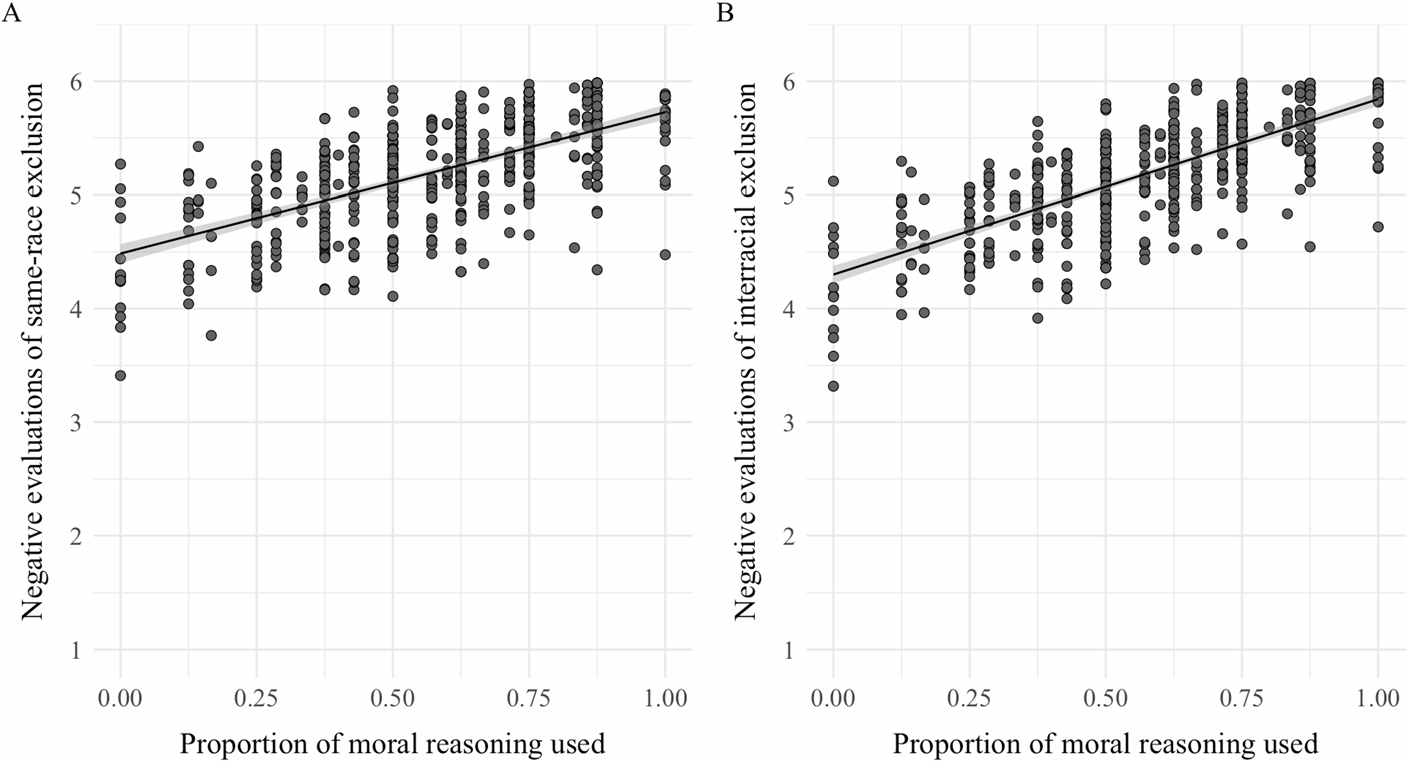
Negative evaluations of exclusion as a function of moral reasoning. *Note*. Greater use of moral reasoning predicted more negative evaluations of both same-race (Panel **A**) and interracial (Panel **B**) exclusion. Both associations were statistically significant (*p* <.001). *N* = 528

In line with our primary hypothesis, moral reasoning was significantly associated with children’s evaluations of exclusion. As illustrated in Fig. 2, greater moral reasoning predicted more negative evaluations of both same-race exclusion, $\:\beta\:$ = 1.07, *B* = 0.21, *SE* = 0.22, *t* = 4.89, *p* <.001, 95% CI [0.64, 1.49], and interracial exclusion, $\:\beta\:$ = 1.40, *B* = 0.27, *SE* = 0.22, *t* = 6.38, *p* <.001, 95% CI [0.97, 1.83]. As predicted, this association was slightly more pronounced in evaluations of interracial exclusion compared to those of same-race exclusion. Additionally, grade differences emerged such that 5th grade participants evaluated same-race exclusion, $\:\beta\:$ = -0.28, *B* = -0.23, *SE* = 0.12, *t* = -2.13, *p* = .03, 95% CI [-0.52, -0.04], and interracial exclusion, $\:\beta\:$ = -0.26, *B* = -0.22, *SE* = 0.12, *t* = -2.25, *p* = .02, 95% CI [-0.50, -0.02], more positively than 3rd grade participants.

### Expectations for inclusion

Contrary to our predictions, children’s use of moral reasoning did not significantly predict their expectations for same-race inclusion, $\:\beta\:$ = 0.15, *B* = 0.03, *SE* = 0.22, *t* = 0.65, *p* = .51, 95% CI [-0.29, 0.58], nor for interracial inclusion, $\:\beta\:$ = 0.09, *B* = 0.01, *SE* = 0.26, *t* = 0.32, *p* = .75, 95% CI [-0.43, 0.60].

### Desire to play with diverse peers

**Fig. 3:**
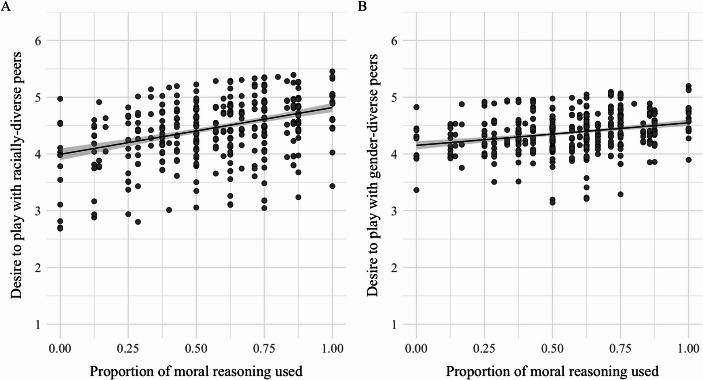
Desire to play with diverse peers as a function of moral reasoning. *Note*. Greater use of moral reasoning predicted a stronger desire to play with racially-diverse peers (Panel **A**; *p* = .03), but not gender-diverse peers (Panel **B**; *p* = .11). *N* = 528

As shown in Fig. 3, greater use of moral reasoning significantly predicted a stronger desire to play with racially-diverse peers, $\:\beta\:$ = 0.48, *B* = 0.11, *SE* = 0.21, *t* = 2.26, *p* = .03, 95% CI [0.06, 0.90]. However, moral reasoning was not significantly associated with a greater desire to play with gender-diverse peers, $\:\beta\:$ = 0.30, *B* = 0.08, *SE* = 0.19, *t* = 1.58, *p* = .11, 95% CI [-0.07, 0.66].

### Empathy toward diverse peers

**Fig. 4:**
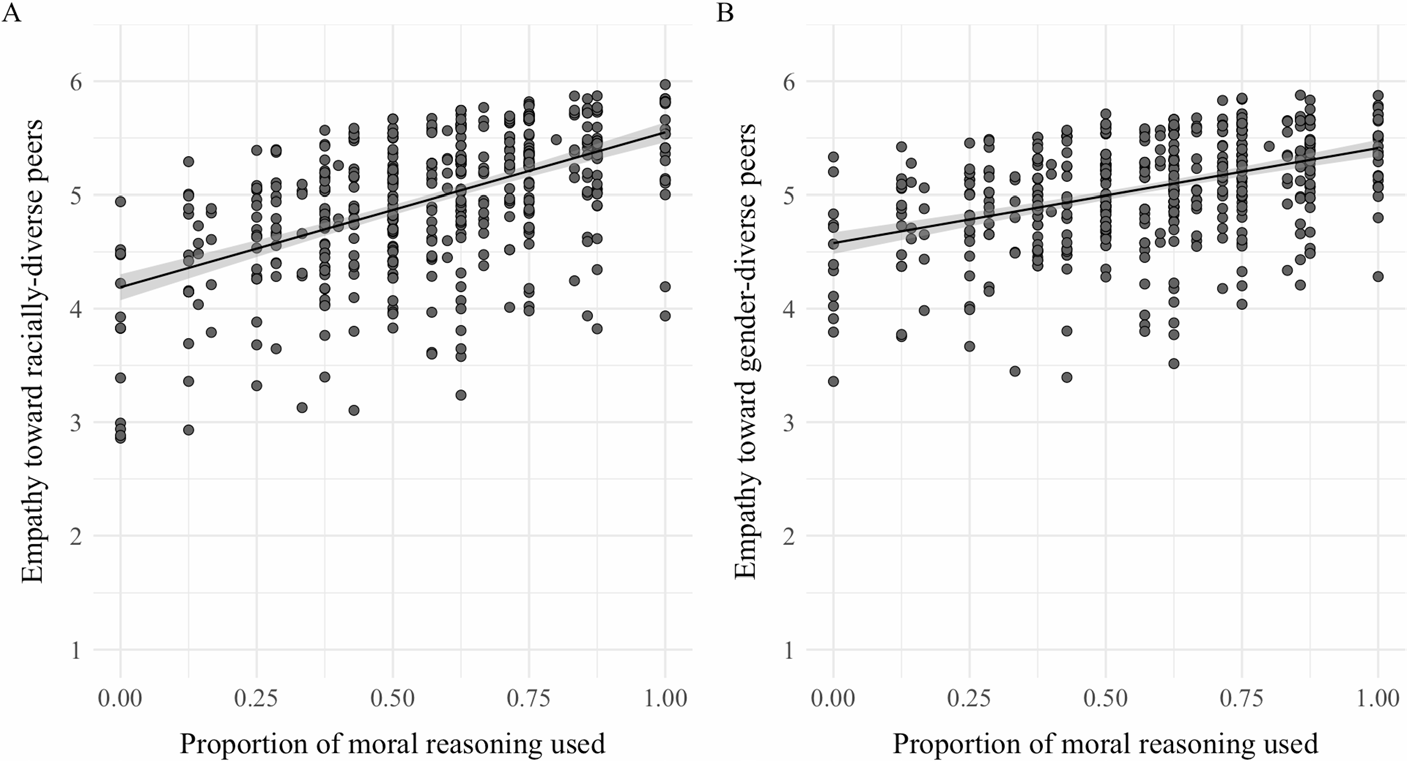
Empathy toward diverse peers as a function of moral reasoning. *Note*. Greater use of moral reasoning predicted greater empathy toward racially-diverse peers (Panel **A**; *p* = .008), but not gender-diverse peers (Panel **B**; *p* = .15). *N* = 528

As depicted in Fig. 4, greater use of moral reasoning significantly predicted greater empathy toward racially-diverse peers, $\:\beta\:$ = 0.61, *B* = 0.12, *SE* = 0.23, *t* = 2.68, *p* = .008, 95% CI [0.16, 1.05]. However, moral reasoning was not significantly related to empathy toward gender-diverse peers, $\:\beta\:$ = 0.33, *B* = 0.07, *SE* = 0.22, *t* = 1.45, *p* = .15, 95% CI [-0.11, 0.76]. Gender differences emerged such that girls exhibited greater empathy toward both gender-diverse peers, $\:\beta\:$ = 0.27, *B* = 0.26, *SE* = 0.10, *t* = 2.72, *p* = .007, 95% CI [0.08, 0.47] and racially-diverse peers, $\:\beta\:$ = 0.31, *B* = 0.27, *SE* = 0.11, *t* = 2.84, *p* = .005, 95% CI [0.10, 0.52], compared to boys.

## Discussion

The present study examined how children’s use of moral reasoning in response to intergroup exclusion scenarios related to their broader inclusive attitudes and behaviors. Across an eight-week structured program designed to foster perspective-taking and inclusion, children who more frequently employed moral reasoning demonstrated stronger inclusive orientations. Specifically, children who used more moral reasoning in the DIY scenarios exhibited greater desire to play with racially-diverse peers, greater empathy for racially-diverse peers, and more negative evaluations of exclusion upon completion of the program. These findings build on prior research linking moral reasoning to inclusive attitudes (Elenbaas et al., 2016; Malti et al., 2009) by demonstrating that consistent engagement in moral reasoning across diverse social contexts can generalize to broader intergroup attitudes and behaviors.

Moral reasoning was more strongly associated with inclusivity in racial contexts compared to gender contexts. For instance, moral reasoning predicted more negative evaluations of interracial exclusion and was linked to greater empathy toward and desire to play with racially-diverse peers, but not gender-diverse peers. Given that gender is particularly salient to children from a young age (Arthur et al., 2008; Bigler & Liben, 2007), children may view gender-based exclusion as more acceptable due to normative gender preferences and may view gender differences as a significant barrier to overcome in terms of being able to relate to one another. The stronger effects observed in racial contexts may reflect heightened moral salience of race-based exclusion, consistent with evidence that children are aware of racial fairness concerns from an early age (Waxman, 2021). Consequently, children may view race as a less legitimate basis for exclusion. Another possibility is that the program’s greater inclusion of scenarios involving racial and ethnic exclusion, compared to the fewer scenarios addressing gender exclusion, contributed to these differential effects. Future research should explore whether balancing the representation of social identity contexts affects the strength of these associations.

Moral reasoning was not significantly associated with children’s expectations for inclusion, in contrast to its associations with other inclusive outcomes. Several factors may explain this discrepancy. While prior work has linked perceived norms of inclusivity to intergroup attitudes (Rutland et al., 2010), expectations about peer behavior may be more heavily influence by social norms and peer group dynamics rather than their individual moral evaluations. While children may personally believe exclusion is wrong, they might also anticipate that peers will behave according to prevailing group preferences or stereotypes. Predicting the behavior of others, particularly in peer settings, may therefore rely less on moral reasoning and more on children’s understanding of group conformity, social hierarchies, or past experiences with peer inclusion (Rutland et al., 2024). Additionally, identifying unjust behaviors such as exclusion may be more straightforward than anticipating proactive inclusive acts, which are often perceived as discretionary rather than obligatory. The structure of the DIY program may have further contributed to this pattern. While the curriculum tool encouraged individual moral reasoning about exclusion scenarios, the teacher-facilitated classroom discussions emphasized group collaboration and positive peer interactions. These discussions may have shaped children’s perceptions of inclusion norms at a broader classroom level, potentially diluting the direct link between individual reasoning and expectations about peer behavior.

Demographic factors, including race, gender, and grade did not moderate the relationship between moral reasoning and inclusivity outcomes. However, we observed main effects of gender and grade on certain outcomes. Fifth graders evaluated exclusion more positively than younger children, consistent with developmental research showing that older children increasingly consider group dynamics and peer norms in their reasoning about exclusion (Killen et al., 2010; Rutland et al., 2024). Gender differences in empathy were also observed, with girls expressing greater empathy toward diverse peers. This aligns with previous findings that girls, as members of a historically disadvantaged group, may develop stronger perspective-taking abilities through their social experiences (Malti et al., 2009; Rizzo & Killen, 2018).

Several limitations should be considered when interpreting these findings. First, moral reasoning was assessed only in the intervention group, precluding direct comparisons with a control group. While prior analyses indicated that DIY and business-as-usual (BAU) participants did not differ at pretest, the current analyses do not permit causal inferences about the program’s impact on moral reasoning. Future studies should incorporate pretest-posttest assessments of moral reasoning across both intervention and control groups to evaluate program effects more rigorously.

Second, the task design limited moral reasoning responses to scenarios where participants judged exclusion as wrong. Although prior research shows that children rarely invoke moral reasoning to justify exclusion as acceptable (Killen et al., 2013), this design feature restricted the contexts in which moral reasoning could be expressed. Nevertheless, the observed variability in participants’ use of moral reasoning when rejecting exclusion suggests that moral reasoning reflects more than simply evaluating exclusion as wrong. Not all participants who judged exclusion as wrong provided moral justifications, underscoring that reasoning and evaluation, while related, are distinct processes. The fact that moral reasoning emerged as a significant predictor of intergroup outcomes highlights the importance of examining how children construct explanations for social exclusion, not merely whether they reject it. Future work should build on these findings by exploring reasoning processes across a wider range of evaluative contexts to disentangle these constructs more fully.

Additionally, while participants engaged in moral reasoning during teacher-led classroom discussions, we did not systematically code or quantify these instances. While students had a finite number of opportunities to use moral reasoning in the DIY tool, they theoretically had unlimited opportunities to do so during the classroom discussions. It remains possible that reasoning articulated in a social, interactive context may be more predictive of inclusive orientations than reasoning captured through individual engagement with the curriculum tool. It is important for future work to investigate how the frequency and depth of moral reasoning throughout the program contribute to outcomes. Further, examining spontaneous moral reasoning during these discussions will help to provide a more ecologically valid understanding of these effects.

Beyond moral reasoning, other mechanisms, such as empathy, perspective-taking, and perceived social norms, likely contribute to the development of inclusive attitudes. Although our study focused on moral reasoning, these additional processes warrant investigation as potential mediators or complementary pathways. For example, prior research has shown that empathy-based interventions are effective in reducing prejudice, particularly when tailored to specific outgroups (Carlo et al., 2022). Future studies should explore how programs like DIY foster inclusivity through multiple mechanisms (e.g., perspective-taking, stereotype reduction, etc.) and whether these pathways interact.

Finally, the repeated engagement with moral reasoning prompts throughout the program raises questions about developmental trajectories of reasoning itself. Longitudinal analyses, such as latent growth curve modeling, could elucidate whether increases in moral reasoning across sessions predict subsequent changes in inclusivity. Alternatively, person-centered approaches (e.g., latent class analysis) could identify subgroups of children who show distinct patterns of reasoning growth or stability, providing a richer understanding of individual differences in program responsiveness.

## Conclusion

In sum, these findings suggest that fostering moral reasoning through structured engagement with intergroup exclusion scenarios can support the development of inclusive orientations in childhood. Programs like DIY, which prompt children to consider fairness and others’ welfare, may strengthen the connection between moral reasoning and inclusive attitudes, intentions, and behaviors across diverse social contexts. While the current results do not directly demonstrate bias reduction, they highlight the potential for moral reasoning to serve as a foundational mechanism for promoting inclusivity.

Importantly, moral reasoning likely operates alongside other processes such as empathy, perspective-taking, and sensitivity to group norms, each contributing uniquely to children’s intergroup attitudes. Future research should explore how these factors interact, examining growth in moral reasoning over time and assessing how these cognitive and social-emotional skills translate into children’s everyday peer interactions.

Enhancing children’s capacity to reason about fairness and inclusion represents a promising avenue for fostering equitable and inclusive peer environments. By equipping children with the tools to recognize and challenge exclusionary behaviors, structured programs that emphasize moral reasoning can contribute meaningfully to efforts aimed at reducing bias and promoting positive intergroup relationships from an early age.
